# Truancy, Psychosocial Distress, and Risk Behaviors in School‐Going Adolescents: Insights From a National School‐Based Survey in the Philippines

**DOI:** 10.1111/josh.70147

**Published:** 2026-04-06

**Authors:** Omid Dadras

**Affiliations:** ^1^ Research Centre for Child Psychiatry University of Turku Turku Finland

**Keywords:** Philippines, psychosocial, social support, substance use, truancy, violence

## Abstract

**Background:**

Truancy, or unexcused school absenteeism, is linked to adolescent psychosocial and behavioral problems and may serve as a behavioral marker of developmental and ecological vulnerability. This study examined associations between truancy and psychosocial distress, violence, limited social support, and substance use among Filipino students.

**Methods:**

Data were drawn from the 2019 Philippines Global School‐Based Student Health Survey (GSHS) of adolescents in Grades 7–10 (ages 13–17). Truancy was defined as missing school without permission during the past 30 days. Twenty‐three variables covering psychosocial problems, violence, social support, and substance use were analyzed using logistic regression, stratified by sex.

**Results:**

About 32.6% of students reported truancy in the past month, with higher odds among older and male adolescents. Truant students had elevated odds of loneliness, anxiety, suicidal behaviors, bullying, violence, and substance use. Female students exhibited higher odds of alcohol and marijuana use relative to males. Truant students were more likely to report limited parental support and peer isolation.

**Implications for School Health Policy, Practice, and Equity:**

Recognizing truancy as an early warning marker can inform school‐based screening, psychosocial support, and gender‐sensitive interventions to reduce inequities.

**Conclusions:**

Truancy may reflect underlying psychosocial challenges, underscoring the need for proactive, tiered school‐based identification and support strategies for at‐risk adolescents.

## 1 Introduction

Adolescence is recognized as a critical developmental period marked by extensive biological, cognitive, emotional and social transitions, which shape trajectories of health, behavior, and social functioning [1, 2]. During this window, adolescents are especially sensitive to environmental stressors, peer influence, and changes in family or school contexts; consequently, early disengagement from school (e.g., truancy) may reflect or precipitate broader psychosocial vulnerabilities [2].

While absenteeism encompasses all types of absences, truancy specifically refers to unauthorized or unjustified absences from school [3]. Truancy or unexcused school absenteeism among adolescents is a multifaceted issue that can have profound implications for behavioral and psychosocial well‐being [4, 5]. While it has traditionally been viewed through an educational lens, recent research has suggested its utility as a potent marker for identifying adolescents at risk of various psychosocial challenges in school‐going adolescents [6, 7].

Studies have shown a strong association between school absenteeism and youth psychopathology, with reciprocal influences between absenteeism and mental health issues [6]. Absenteeism is linked to psychological disorders like anxiety, depression, conduct problems, and social phobia, indicating a complex interplay between school avoidance and psychopathology [8, 9, 10]. Additionally, unexcused absenteeism or truancy has been found to be associated with more severe psychosocial risks, including suicidal behaviors and substance use disorders, with far‐reaching consequences for adolescents and their families [10]. The presence of these disorders can significantly impact a student's ability to attend school regularly and can have long‐term consequences if not addressed [11]. Therefore, early recognition and intervention through the lens of truancy can have tangible implications addressing school avoidance and its associated psychopathologies.

The Philippines exhibits notably high truancy rates among adolescent students compared to neighboring nations. National survey data indicate that more than 30% of Filipino students aged 13–15 years reported truancy in the past 30 days [12], placing the Philippines above the ASEAN regional average of 24.8% for past‐month truancy [10]. This pattern of unauthorized absence, distinct from excused or illness‐related absences, appears across multiple educational levels, with localized school studies documenting chronic absenteeism rates ranging from 39% to 50% among identified at‐risk students [13, 14]. In comparison, global data show considerable variation in truancy rates; for example, Laos reported a 40.7% prevalence of truancy in the past 30 days [15]. Meta‐analytic evidence further suggests that a range of internalizing and externalizing problems such as anxiety, substance use, poor parental involvement, and negative school climate are consistently associated with truancy across diverse settings [16, 17]. Within the Philippine context specifically, studies point to financial strain, family work obligations, transportation barriers, low parental involvement, health concerns, unfavorable school environments, and academic overload as major contributors to truancy behavior [18, 19].

Given that education plays a central role in adolescent development and long‐term human capital formation, understanding how absenteeism intersects with adolescents' psychosocial vulnerabilities is crucial [20]. Despite evidence linking truancy with psychosocial risk factors, relatively little is known about how these relationships manifest among Filipino adolescents. This gap is concerning in light of increasing reports of depression, suicidal behavior, and violence among adolescents in recent years [12, 21]. Examining truancy as a potential early indicator of such vulnerabilities may therefore offer valuable insights for policymakers and help inform timely, targeted interventions. Conceptually, this study reframes truancy as a behavioral marker of accumulated psychosocial vulnerability, rather than solely an educational or disciplinary concern. Drawing on developmental psychopathology and ecological systems perspectives [22, 23], we position truancy as an observable manifestation of underlying emotional distress, exposure to adverse environments, and limited social support across family, peer, and school contexts. Meta‐analytic and cross‐national studies have shown that internalizing symptoms (e.g., loneliness, anxiety, suicidal behaviors), exposure to violence and bullying, limited parental and peer support, and substance use are among the most robust and recurrent correlates of truancy in adolescents [4, 10, 16].

Against this background, using nationally representative data from Filipino secondary‐school students, we examined the prevalence of truancy and estimated the odds of multiple psychosocial risk domains including internalizing symptoms, violence exposure, limited social support, and substance use among truant versus non‐truant adolescents. By integrating these domains within a single, large‐scale dataset, this study advances current knowledge by conceptualizing truancy as a multidimensional indicator of adolescent well‐being, thereby informing intervention strategies that bridge educational engagement and mental health within adolescents' social ecologies.

## 2 Methods

### 2.1 Study Setting and Design

The 2019 Philippines Global School‐Based Student Health Survey (GSHS) was conducted among students in Grades 7–10 in Philippines schools, typically encompassing ages 13–17. According to the WHO Global School‐based Student Health Survey documentation, the sampling frame included secondary schools across all major regions of the Philippines, including Luzon, Visayas, and Mindanao [12]. As such, the survey reflects a mix of urban and rural schools as well as both public and private secondary schools nationwide. However, detailed school‐level identifiers (e.g., school name, type, or specific location) are not included in the public‐use dataset; therefore, the present analysis could not stratify results by school characteristics. Utilizing a two‐stage cluster sample design, the survey aimed to gather data representative of all students in these grade levels across the Philippines. In the initial stage, schools were chosen with a probability proportional to their enrollment size, while in the subsequent stage, classes were randomly selected, and all students in the chosen classes were eligible for participation. The Philippines GSHS covered various aspects, including alcohol use, dietary behaviors, drug use, hygiene, mental health, physical activity, protective factors, violence, and unintentional injury. Student responses were self‐reported via computer‐scannable answer sheets. The survey achieved a 100% school response rate, with a student response rate of 85%, resulting in an overall response rate of 85%. A total of 10,149 students participated in the survey, with prevalence estimates (percentages) and 95% confidence intervals provided in the Results section.

### 2.2 Study Variables

#### 2.2.1 Independent Variable

*Truancy* was measured using the standard GSHS item: “During the past 30 days, on how many days did you miss classes or school without permission?.” Responses were categorized as 0 days (no truancy) and 1 or more days (any truancy), consistent with previous GSHS analyses [15]. Truancy was assessed based on the standard GSHS past‐30‐day item and reflects recent (past‐30‐day) unexcused absenteeism rather than chronic absenteeism.

#### 2.2.2 Dependent Variables

Based on a comprehensive literature review, 23 relevant variables were selected and extracted from the Philippines GSHS 2019 dataset. We acknowledge that dichotomizing ordinal variables reduces response variability and may overlook graded differences. However, in GSHS research, dichotomization is commonly used [10, 15, 24], particularly in low‐ and middle‐income country (LMIC) contexts, because some upper‐frequency categories contain very small cell counts that produce unstable regression estimates. Given the relatively low frequency of “Most of the time/Always” responses for several psychosocial and violence‐related items in this dataset, dichotomization was necessary to ensure stable estimates and appropriate sample size per category. This approach also allowed comparability with prior GSHS studies across regions. Demographic variables included age (≤ 14 vs. > 14 years), sex (male or female), and grade level (7th–10th). Detailed variable definitions and coding are presented in Table 1.

**TABLE 1:** Description and coding of dependent variables.

Domain	Variable	GSHS item (summary)	Coding
Psychosocial problems	Loneliness	Felt lonely (past 12 months)	1 = always/often/sometimes; 0 = never/rarely
	Anxiety	Worried so much could not sleep (past 12 months)	1 = always/often/sometimes; 0 = never/rarely
	Suicidal ideation	Seriously considered suicide (past 12 months)	1 = yes; 0 = no
	Suicide attempt	Attempt suicide (past 12 months)	1 = yes; 0 = no
Violence‐related	Bullied at school	Bullied on school property (past 12 months)	1 = yes; 0 = no
	Bullied outside school	Bullied off school property (past 12 months)	1 = yes; 0 = no
	Cyberbullied	Cyberbullied via social media (past 12 months)	1 = yes; 0 = no
	Physically attacked	Physically attacked (past 12 months)	1 = ≥ 1 times; 0 = 0 time
	Physical fight	Involved in physical fight (past 12 months)	1 = ≥ 2 times; 0 = 0 times
Limited social support	Parental neglect	Parents checked homework (past 30 days)	1 = always/often; 0 = never/rarely/sometimes
	Parental disengagement	Parents understood problems (past 30 days)	1 = always/often; 0 = never/rarely/sometimes
	Parental detachment	Parents knew free‐time activities (past 30 days)	1 = always/often; 0 = never/rarely/sometimes
	Peer isolation	Number of close friends	1 = ≥ 1 friend; 0 = none
	Peer rejection	Students kind/helpful at school (past 30 days)	1 = always/often; 0 = never/rarely/sometimes
	Food insecurity	Went hungry due to lack of food (past 30 days)	1 = always/often/sometimes; 0 = never/rarely
Substance use	Current marijuana use	Marijuana use (past 30 days)	1 = ≥ 1 times; 0 = 0 time
	Current alcohol use	≥ 1 alcoholic drink (past 30 days)	1 = yes; 0 = no
	Ever drunk	Ever drank until drunk	1 = ≥ 1 time; 0 = never
	Drug use < 14 years	Age at first drug use	1 = < 14 years; 0 = ≥ 14/never
	Alcohol use < 14 years	Age at first alcohol use	1 = < 14 years; 0 = ≥ 14/never

### 2.3 Statistical Analysis

Descriptive statistics were used to describe the demographic characteristics of the participants. Logistic regression, adjusting for age distribution, was used to examine the odds of the dependent variables, including the demographic characteristics, psychosocial problems, violence exposure, limited social support, and substance use for those with truancy. Separate analyses were conducted for male and female participants to explore potential differences in these associations between sexes. Multivariable models were adjusted for age because the public‐use GSHS dataset does not include socioeconomic indicators, academic grades, nor identifiable school‐level contextual variables such as school type or urban/rural location. Therefore, additional potential confounders could not be included. This limitation is common to secondary analyses of publicly available GSHS data. The results were graphically represented using forest plots, presenting the odds ratios (OR) alongside their respective 95% confidence intervals (95% CI). The effect of the multi‐stage sampling design and sampling weight due to disproportionate sampling was accounted for in all analyses. The analysis was performed in STATA 17 (Stata Corporation, College Station, Texas, USA) and the statistically significant level was determined as 0.05.

## 3 Results

The analytic sample included 10,149 school‐going Filipino adolescents from the 2019 GSHS, with approximately equal representation of males and females; most participants were aged 14 years or younger. The overall prevalence of past‐month truancy was 32.6%.

Truancy was more prevalent among adolescents older than 14 years (35.8%) compared with those aged 14 years or younger (30.9%), corresponding to 25% higher odds of truancy among older adolescents (OR 1.25, 95% CI 1.07–1.46). Male adolescents reported a higher prevalence of truancy (39.2%) than females (25.8%). Females had significantly lower odds of truancy compared with males (OR 0.54, 95% CI 0.49–0.60). No significant differences in truancy prevalence were observed across grade levels.

### 3.1 The Odds of Psychosocial Problems Among Truant Adolescents

**FIGURE 1:**
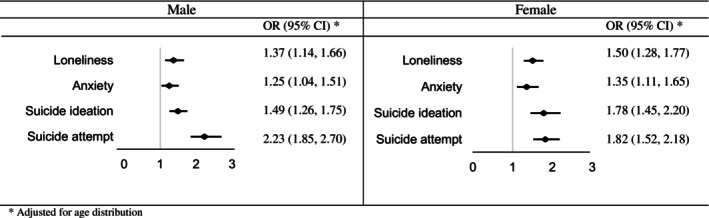
The odds of psychosocial problems among students with truancy.

As Figure 1 shows, the odds of experiencing all psychosocial problems were notably higher among both male and female adolescents with truancy. For male adolescents, the odds ratios ranged from 1.25 for anxiety to 2.23 for suicide attempt; likewise, for female adolescents, the odds ranged from 1.35 for anxiety to 1.82 for suicide attempt.

### 3.2 The Odds of Violent Behaviors Among Truant Adolescents

**FIGURE 2:**
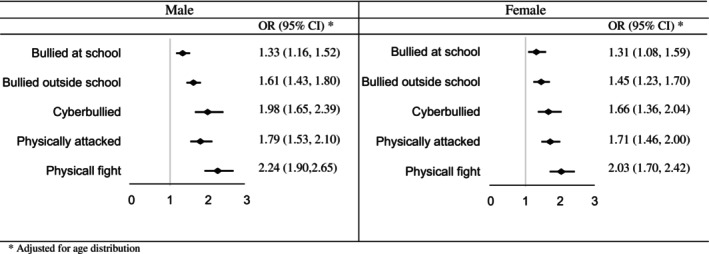
The odds for violent behaviors among students with truancy.

As displayed in Figure 2, the odds of experiencing violent behaviors were consistently and significantly higher among truant adolescents. For males, the odds of being bullied at school, outside school, and cyberbullied were respectively 33%, 61%, and 98% higher among truant adolescents. Additionally, the odds of experiencing physical attacks and engaging in physical fights were 1.79 and 2.24 times higher for truant adolescents. Similarly, among females, the odds of violence were significantly higher among truant adolescents, with odds ratios of 1.31 for being bullied at school, 1.45 for being bullied outside school, 1.66 for cyberbullying, 1.71 for experiencing physical attacks, and 2.03 for engaging in physical fights.

### 3.3 The Odds of Limited Social Support Among Truant Adolescents

**FIGURE 3:**
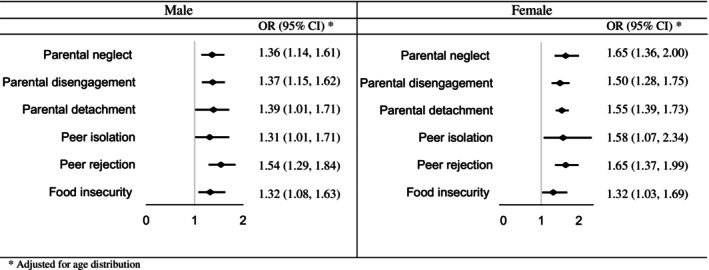
The odds for limited social support among students with truancy.

As Figure 3 illustrates, the odds were significantly higher across both limited parental and peer support domains in both male and female adolescents with truancy. For males, the odds of parental neglect, parental disengagement, and parental detachment were 36%, 37%, and 39% higher among truant adolescents, respectively. Additionally, the odds of peer isolation and rejection were 1.31 and 1.54 times higher among truant adolescents. Similarly, for females, the odds of parental neglect, parental disengagement, and parental detachment were 65%, 50%, and 55% higher among truant adolescents, respectively. Female adolescents also demonstrated increased odds of peer isolation (OR = 1.58) and peer rejection (OR = 1.65).

### 3.4 The Odds of Substance Use Among Truant Adolescents

**FIGURE 4:**
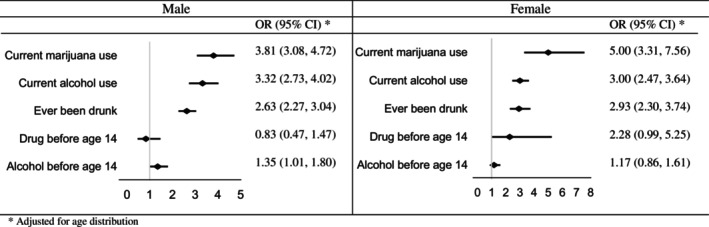
The odds of substance use among students with truancy.

Figure 4 displays the odds of substance use among male and female adolescents with truancy. For males, the odds were notably elevated for marijuana use (OR = 3.81), alcohol consumption (OR = 3.32), and having ever been drunk (OR = 2.63) among truant adolescents. However, the odds of drug use before the age of 14 and alcohol use before the age of 14 did not show significant associations. In contrast, female adolescents with truancy exhibited higher odds across all substance use, with 5‐ and 3‐times higher odds for marijuana and alcohol use. Additionally, they had increased odds of having ever been drunk (OR = 2.93) and drug use before the age of 14 (OR = 2.28), although the latter did not reach statistical significance. However, similar to males, the odds of alcohol use before the age of 14 were not significantly elevated.

## 4 Discussion

Approximately 32.56% of Filipino adolescents attending school reported unexcused absences or truancy within the past month, a notably higher rate compared to neighboring countries such as Thailand, Indonesia, and Vietnam, where approximately 20% of adolescents reported truancy during the same period [25, 26, 27]. Various factors could contribute to this discrepancy, including socio‐economic conditions, cultural influences, educational policies, and the availability of support systems for students [28]. Additionally, differences in the enforcement of truancy laws and the accessibility of educational resources may also play a significant role. The study revealed notable disparities in truancy prevalence based on age and sex, with older adolescents and males exhibiting higher rates of truancy. It is important to note that truancy may also reflect structural and contextual barriers, including economic hardship, transportation difficulties, household responsibilities, or perceptions of school safety [18, 29, 30], rather than solely psychosocial or behavioral risk. In the Philippine context, factors such as persistent poverty, regional inequality in educational access, and variability in school infrastructure and resources may shape school attendance and contribute to absenteeism among adolescents [18, 30]. Because the GSHS measure captures “missing classes without permission” but does not identify the reasons for absence, we cannot distinguish between disengagement, avoidance, or contextual constraints. Therefore, interpretations should be cautious, and further research incorporating socioeconomic indicators and school‐level factors is needed to disentangle competing explanations for truancy.

These findings align with previous research emphasizing age and gender as significant predictors of school attendance [10, 24]. Older adolescents, especially around age 15, are prone to truancy due to heightened social pressures, academic challenges, and a desire for independence. Moreover, greater autonomy and reduced parental supervision in this age group make skipping school easier without immediate repercussions [31]. These factors, coupled with societal expectations and norms that place less emphasis on academic performance for males compared to females, may be linked to a higher tolerance for school absenteeism among boys [32]. Sex differences in these associations likely reflect both developmental patterns in emotion and behavior and context‐specific gender norms in the Philippines. Adolescent boys more often show externalizing behaviors such as aggression and rule‐breaking, whereas girls are more likely to report internalizing symptoms like anxiety, sadness, and loneliness, aligning with broader evidence on gendered emotion expression and psychopathology [33, 34, 35]. In addition, age and gender differences in truancy patterns may map onto developmental shifts in identity work and social expectations across early to late adolescence, as well as gendered norms around autonomy, risk‐taking, and affiliation. From Erikson's perspective [36], adolescence is characterized by the psychosocial task of “identity versus role confusion,” during which young people actively explore social roles, values, and peer affiliations. Truancy in this context may therefore reflect ongoing identity exploration (e.g., peer conformity, autonomy seeking) as well as role confusion, particularly among adolescents who perceive school environments as inconsistent with their developing self‐concepts. Thus, age and gender differences observed in this study may be partly understood as developmental variations in how adolescents negotiate identity formation, autonomy, and belonging during the school years.

The findings of the present study indicated higher odds of loneliness, anxiety, suicidal ideation, and suicide attempt among truant students. Previous studies have also observed higher rates of isolation and mental distress among truant students, which can contribute to a higher risk of suicidal behavior [37, 38]. Loneliness, anxiety, and other mental health issues can drive truancy, creating a cycle where avoidance of school exacerbates existing mental health challenges [31]. Truant students may experience a decline in school performance, loss of friendships, decreased self‐esteem, and increased mental health problems, ultimately linking to a higher likelihood of suicidal ideation and attempts [39]. In addition, the heightened odds of being bullied at or outside school, as well as being cyberbullied among truant students, underscore the vulnerability of this population to peer victimization. The psychological distress experienced by victims of bullying, whether traditional or cyber, is significantly elevated and can put them at higher risk of experiencing depression, anxiety, loneliness, suicidal ideations, truancy, lower academic achievement, and dropping out of school [40, 41, 42, 43]. Moreover, truant students may face challenges in forming positive relationships with peers, which can further exacerbate their risk of being bullied [44]. That being said, truancy can serve as a valuable marker for underlying psychosocial problems contributing to depression, suicidal behaviors, and victimization experiences in students. Recognizing the significance of truancy is vital for designing effective interventions and support systems to prevent the associated negative consequences, not only in the Philippines but also in other contexts.

In the present study, the odds of being physically attacked and engaging in physical fights were higher among students with truancy, indicating a heightened risk of violence among truant adolescents. Chronic truancy is often linked to delinquency, drug use, and violent behavior [45, 46]. Research indicates that adolescents who engage in violent behavior are more likely to repeat it, along with those who use drugs or join gangs [46]. While persistent truancy has been associated with later delinquency in other studies [47]. The present findings reflect associations within adolescence only and cannot address long‐term trajectories. These results nevertheless suggest that truancy may signal broader behavioral risks that warrant early identification and supportive school‐based responses.

Truant adolescents in this study were more likely to report parental neglect, disengagement, and detachment. Given the critical role of both parental and peer factors in determining the level of social support available to individuals, this underscores the significance of parental involvement and support in mitigating the adverse effects of truancy on adolescents' social networks [48]. This is more pronounced in adolescents who prioritize social goals over academic achievement, who can be influenced by social support in addressing inattention and lack of class engagement [49, 50]. Similarly, peer dynamics significantly influence social support outcomes and school engagement [50], with truant Filipino adolescents exhibiting higher odds of peer isolation or rejection. The literature suggests that social support from friends, especially during adolescence, can be more critical than family support in reducing the likelihood of school absenteeism and improving academic outcomes [49]. Therefore, social support can play a crucial role in addressing inattention and lack of school engagement, particularly in adolescents who prioritize social goals over academic achievements [51]. Conversely, truancy can indicate limited social support among school‐going adolescents and can provide valuable insights into family and peer dynamics of truant adolescents. These insights are particularly important for students with chronic truancy and should be considered in the counseling and support systems for this group.

### 4.1 Implications for School Health Policy, Practice, and Equity

This study underscores truancy as both an educational and public health concern, warranting its use as a routine marker for psychosocial distress, violence, and risky behaviors. In several high‐income settings such as Australia [52, 53], the United States [54, 55], and the United Kingdom [56], school attendance policies integrate early identification systems, routine screening, and referral pathways to school counselors and multidisciplinary support teams; these models have demonstrated effectiveness in reducing absenteeism and identifying at‐risk students. Evidence‐based frameworks such as Multi‐Tiered Systems of Support (MTSS) highlight the benefit of combining universal prevention (e.g., social–emotional learning and bullying prevention), targeted counseling, and intensive case‐management for students with persistent absenteeism [54, 55]. Adapting similar tiered approaches to the Philippine context, including integration into existing guidance counselor roles and community‐based services rather than reliance on specialized staffing, may be a feasible and resource‐appropriate strategy. Strengthening parental engagement, peer support, and gender‐sensitive programming could further address vulnerabilities, while ensuring access to mental health services, anti‐bullying initiatives, and substance‐use prevention through school–family–community collaboration, particularly in underserved settings.

### 4.2 Limitations

Although the findings suggest that truancy may be a useful indicator of underlying psychosocial problems, violence, limited social support, and substance use among Filipino students, some limitations should be considered when interpreting the results. First, the study employed a cross‐sectional design, which precludes causal inference, and reliance on self‐reported data, which may be subject to recall and social desirability biases, particularly for sensitive behaviors such as truancy, violence, and substance use. Although the GSHS uses anonymous questionnaires to reduce these biases, misclassification of some behaviors cannot be ruled out. Additionally, truancy was measured over the past 30 days only, which limits the ability to differentiate episodic from chronic patterns of absenteeism. Therefore, our measure predominantly reflects recent absenteeism rather than long‐term school disengagement. Therefore, future investigations should account for this extended timeframe to provide a more comprehensive understanding of the effects of truancy on adolescent well‐being. Because school‐level identifiers were anonymized in the public‐use dataset, we were unable to examine differences by school type, school resources, or urban versus rural settings. The GSHS is designed as a surveillance and monitoring instrument rather than a full socioeconomic dataset; therefore, common confounders such as household income, parental education, academic achievement, or school characteristics are not available in the public‐use file. Therefore, analyses could not control for several potential confounders such as family income, school type, or academic performance. Consequently, residual confounding cannot be ruled out. Future studies using datasets that include socioeconomic and contextual variables could provide a more comprehensive understanding of these associations. Moreover, because the GSHS item does not capture the reasons for missing school, truancy in our study may reflect structural constraints (e.g., poverty, transportation, unsafe environments) rather than only psychosocial or behavioral factors. Therefore, contextual explanations cannot be ruled out. Future research incorporating socioeconomic measures and regional educational indicators could help disentangle the relative contribution of structural versus psychosocial determinants of truancy in the Philippines. We also note that dichotomization may reduce nuance in the original ordinal responses. Future studies may benefit from modeling variables with more granular categories or continuous approaches where sample sizes permit.

## 5 Conclusion

The findings suggest that truancy may serve as an important early warning sign of underlying psychosocial difficulties, exposure to violence, and risk behaviors including substance use. While these patterns represent associations rather than causal pathways, recognizing truancy as a potential indicator can inform targeted screening and school‐based interventions aimed at supporting adolescents who may be experiencing psychosocial vulnerability. Strengthening early identification systems, guidance counseling, and school–family–community collaboration may help mitigate associated risks and improve student wellbeing. Future research employing longitudinal designs and broader measures of absenteeism is warranted to clarify developmental trajectories and guide effective intervention strategies.

## Funding

The author has nothing to report.

## Ethics Statement

The GSHS 2019 received ethics approval from the pertinent national ethics committee in the Philippines, ensuring adherence to ethical standards. Given that our study constitutes a secondary analysis of the existing data, additional ethical approval was not required.

## Consent

Prior to participation, written informed consent was obtained from either the participants themselves or their guardians.

## Conflicts of Interest

The author declares no conflicts of interest.

## Data Availability

The Philippines GSHS 2019 is a publicly available dataset and is available on the World Health Organization NCD Microdata Repository website at https://extranet.who.int/ncdsmicrodata/index.php/catalog.
